# Synthesis, Characterization, Antioxidant, and Antibacterial Studies of Some Metal(II) Complexes of Tetradentate Schiff Base Ligand: (4E)-4-[(2-{(E)-[1-(2,4-Dihydroxyphenyl)ethylidene]amino}ethyl)imino]pentan-2-one

**DOI:** 10.1155/2015/890734

**Published:** 2015-05-17

**Authors:** Ikechukwu P. Ejidike, Peter A. Ajibade

**Affiliations:** Department of Chemistry, Faculty of Science and Agriculture, University of Fort Hare, Private Bag X1314, Alice 5700, South Africa

## Abstract

Co(II), Ni(II), Cu(II), and Zn(II) complexes of (4*E*)-4-[(2-{(*E*)-[1-(2,4-dihydroxyphenyl)ethylidene]amino}ethyl)imino]pentan-2-one have been synthesized and characterized by elemental analyses, molar conductance, electronic and IR spectral studies, and XRD. FTIR confirmed the ligand coordinates the metal ion to form mononuclear complex via the oxygen and nitrogen atoms of the phenolic group and azomethine group, respectively. Tetrahedral geometry is proposed for Co(II) complex and square-planar geometry for Ni(II) and Cu(II) complexes. The antibacterial studies of the compounds were determined and they show that the metal complexes are more active than the free ligands. The antioxidant activity by DPPH and ABTS method was examined and it shows Cu(II); IC_50_ = 2.31 ± 1.54 *µ*M for DPPH and Co(II); IC_50_ = 1.83 ± 1.08 *µ*M for ABTS were the most active.

## 1. Introduction

In biological processes, inorganic compounds play critical roles and it has been established that many organic compounds used in medicine are activated or biotransformed by metal ions metabolism [1]. Schiff bases are classified as organic ligands derived from the condensation reactions of primary or secondary amines and corresponding aldehydes or ketones (RCH=NR, where R and R represent alkyl/or aryl substitutes) [2]. Schiff bases are considered a very important class of organic ligands possessing diverse applications [3, 4]. Copious transition metal complexes with polydentate Schiff bases containing nitrogen, oxygen, or sulphur donor atoms contribute immensely in biological systems [5, 6]. These complexes exhibit applications in clinical, analytical, and industrial processes [7]. Studies on transitional metal compounds of Schiff base ligands have been of great significance due to their spectral properties and wide applications [8, 9]. These complexes are used as model molecules for biological oxygen carrier systems [3].

Tetradentate Schiff base complexes have been shown to form stable complexes, with coordination taking place through the dinitrogen-dioxygen donor atoms [10]. Zishen et al. [11] have reported that Schiff base complexes derived from 4-hydroxysalicylaldehyde and amines have strong anticancer activity against* Ehrlich ascites carcinoma* (EAC). Schiff bases of ketones, their derivatives, and metal complexes are active as anticancer and antioxidative agents [12–14]. Shelke et al. [15] reported the synthesis of unsymmetrical tetradentate Schiff base ligand: 4-hydroxy-3-(1-{2-(2-hydroxybenzylidene)-amino-phenylimino}-ethyl)-6-methyl-pyran-2-ones and its rare earth metals. The complexes showed enhanced antimicrobial activity compared to the free ligand [15]. Comparatively, little is known about the unsymmetrical Schiff base ligands and their metal(II) complexes with heterocyclic aromatic bases [16]. The oxidative damage caused by ROS on lipids, proteins, and nucleic acids plays a significant role in the development of life limiting chronic diseases such as cancer, hypertension, cardiac infarction, arteriosclerosis, rheumatism, and cataracts [17]. Antioxidants are considered important nutraceuticals on account of their many health benefits and are widely used in the food industry [18, 19].

In our effort towards the development of metal-based therapeutic agents, we present the synthesis, characterization, and biological studies of Co(II), Ni(II), Cu(II), and Zn(II) complexes containing unsymmetrical tetradentate Schiff base ligand: (4E)-4-[(2-{(E)-[1-(2,4-dihydroxyphenyl)ethylidene]amino}ethyl)imino]pentan-2-one derived from ethylene-1,2-diamine, 2′,4′-dihydroxyacetophenone, and 2,4-pentanedione.

## 2. Materials and Methods

### 2.1. Materials

All the chemicals and solvents used were of analytical grade: 2,4-pentanedione from Fluka; ethylenediamine, ascorbic acid, and Zn(II)/Co(II)/Cu(II)/Ni(II) acetate from Merck; and 2′,4′-dihydroxyacetophenone from Aldrich, and were used as received. 1,1-Diphenyl-2-picrylhydrazyl (DPPH), 2,2′-azinobis-3-ethylbenzothiazoline-6-sulfonic acid (ABTS), rutin hydrate, and butylated hydroxytoluene (BHT) were purchased from Sigma Chemical Co. (St. Louis, MO, USA).

### 2.2. Physical Measurements

The elemental analysis of each sample was performed on PerkinElmer elemental analyzer. IR spectra were recorded in KBr with a FT-IR spectrophotometer, PerkinElmer Paragon 2000, in the 4000–400 cm^−1^ region. The molar conductance of the complex in DMF was measured at room temperature using Crison EC-Meter Basic 30+ conductivity cell. Electronic absorption spectra of the ligand and metal complexes were recorded on a PerkinElmer Lambda-25 UV-Vis spectrometer using DMF as solvent in the range 200–800 nm. Powder X-ray diffraction (XRD) patterns were recorded with a Bruker AXS D8 Advance powder X-ray diffractometer (X-ray source: Cu, wavelength 1.5406 Å).

### 2.3. Synthesis of the Ligand: (4E)-4-[(2-{(E)-[1-(2,4-Dihydroxyphenyl)ethylidene]amino}ethyl)imino]pentan-2-one (H_2_LL)

**Scheme 1:**
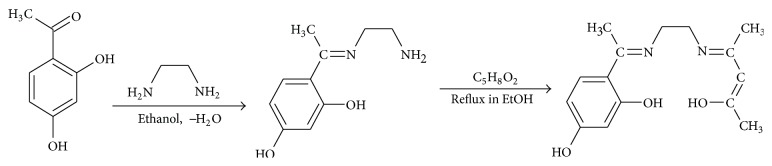
Synthesis of Schiff base ligand (H_2_LL).

The ligand was prepared by a reported method [16]. A typical procedure for the synthesis of Schiff base was as follows. Ethylenediamine (0.015 mol, 0.902 g) in 30 mL ethanol was slowly added to ethanol solution (40 mL) containing 2′,4′-dihydroxyacetophenone (0.015 mol, 2.282 g), followed by the slow addition of acetylacetone (0.015 mol, 1.502 g) dissolved in 30 mL ethanol. The resulting colored mixture was refluxed with stirring for 4 h and cooled and the resulting precipitate was filtered and washed with ethanol, followed by recrystallization in ethanol (yield = 2.53 g, 61.23%) (see Scheme 1).

### 2.4. General Procedure for the Preparation of the Complexes

The complexes were prepared by the addition of 1.5 mmol of Zn(CH_3_COO)_2_·2H_2_O; Cu(CH_3_COO)_2_·2H_2_O; Ni(CH_3_COO)_2_·4H_2_O; or Co(CH_3_COO)_2_·4H_2_O dissolved in about 30 mL of 40% ethanol (v/v) solution, into a hot ethanolic solution (30 mL) (1.5 mmol, 0.4145 g) of H_2_LL in molar ratio (1 : 1). The color of the complexes changed in a few minutes. The resulting mixture was refluxed for 2 h. The precipitated solids were filtered off from the reaction mixture, thoroughly washed with ethanol and then with diethyl ether, and dried over anhydrous calcium chloride.

### 2.5. Antioxidant Assay

#### 2.5.1. Scavenging Activity of 1,1-Diphenyl-2-picrylhydrazyl (DPPH) Radical

DPPH (1,1-diphenyl-2-picryl-hydrazyl) radical scavenging activity evaluation is a standard assay in antioxidant activity studies. It is a rapid technique for screening the radical scavenging activity of specific compounds [19]. The free radical scavenging effects of all the compounds and ligand with DPPH radical were evaluated with various concentrations (100, 200, 300, 400, and 500 *μ*g/mL) of the test compound in 1 mL DMF and were added to 1.0 mL of 0.4 mM methanol solution of DPPH and were stirred thoroughly. After 30 min incubation period at room temperature, the scavenging ability determines the antiradical power of an antioxidant by measuring the decrease in the absorbance of DPPH at 517 nm. Rutin and ascorbic acid (vitamin C) are used as standard drugs. Resulting from a color change, the absorbance decreased when the DPPH is scavenged by an antioxidant, through donation of hydrogen to form a stable DPPH molecule. All tests samples were performed with three replicates to obtain mean ± S.D. The percent of inhibition (*I*%) of free radical production from DPPH was calculated by using the following equation:

(1) DPPH scavenging ability (%)=Abscontrol−AbssampleAbscontrol×100.

#### 2.5.2. ABTS: 2,2′-Azinobis-3-ethylbenzothiazoline-6-sulfonic Acid Radical Scavenging Assay

ABTS scavenging ability of the metal compounds and H_2_LL was studied using the literature procedure [20]. The working solution was prepared by mixing two stock solutions of 7 mM ABTS solution and 2.4 mM potassium persulfate solution in equal amounts (1 : 1) and the solution was allowed to react in the dark for 12 h at room temperature. The resulting solution was further diluted by mixing 1 mL ABTS^+^ solution to obtain an absorbance of 0.706 ± 0.001 units at 734 nm using the spectrophotometer. Test samples (1 mL) were allowed to react with 1 mL of the ABTS^+^ solution, followed by the absorbance reading at 734 nm after 7 min using the spectrophotometer. The ABTS scavenging capacity of the M(II) compounds and Schiff base ligand was compared with that of rutin and butylated hydroxyl toluene (BHT) (standard drugs). All tests and analyses were run in triplicate and the results obtained were averaged. The percentage inhibition was calculated as ABTS radical scavenging activity using the following equation:

(2) $$\begin{array}{c} \text{(\%) Inhibition}=\frac{{\text{Abs}}_{\text{control}}-{\text{Abs}}_{\text{sample}}}{{\text{Abs}}_{\text{control}}}\times 100, \end{array}$$

where Abs_control_ is the absorbance of ABTS radical + DMF and Abs_sample_ is the absorbance of ABTS radical + sample [test samples/standard].

### 2.6. *In Vitro* Antimicrobial Studies

The* in vitro* biological screening effects of the ligand and its metal complexes were tested against 6 bacteria strains consisting of three Gram-positive bacteria, namely,* Staphylococcus aureus* (ATCC 25923),* Streptococcus faecalis* (ATCC 29212), and* Bacillus cereus* (ATCC 10702), and three Gram-negative bacteria, namely,* Pseudomonas aeruginosa* (ATCC 19582),* Escherichia coli* (ATCC 25922), and* Shigella flexneri* (KZN). Ciprofloxacin and Amoxicillin were used as the standard antibacterial agents. Antibacterial activity of the samples was determined by the agar well diffusion method [21]. The bacteria isolates were subcultured on nutrients agar (SAARCHEM, Gauteng SA) plates and incubated at 37°C for 24 h. A loop full of bacteria cells from the nutrient agar plates was incubated into 50 mL of a nutrient broth in a 250 mL sidearm Erlenmeyer flask and incubated at 37°C for 16 h with vigorous shaking. After incubation, the culture was diluted with fresh media to give D_600 nm_ of 0.1 [22]. One hundred microliters of the culture cells was added onto the plate and spread into a bacterial lawn using a sterile glass spreader.

The minimum inhibitory concentration (MIC) of H_2_LL and its metal complexes was determined using agar dilution method as described by the National Committee for Clinical Laboratory Standards (2004) [23]. The bacterial strains were grown at 37°C overnight and maintained on nutrient agar. Inoculums of the test organisms were prepared in normal saline (9 gL^−1^) compared with 0.5 McFarland standard to achieve 5 × 10^5^ (CFU mL^−1^). The suspension was used to inoculate sterile petri plates of 9.0 cm diameter in which the test organisms were grown. A stock solution of the compounds was prepared in DMSO (Sigma) and further diluted in MHB agar at 50°C to give final concentrations ranging from 0.312 to 10 mg/mL; after pouring into plates and allowing the agar to set, plates were inoculated with standardized inocula of the test bacteria. The pates were further incubated at 37°C for 24 h under aseptic conditions. The MIC was recorded as the lowest concentration at which no visible growth was observed.

## 3. Results and Discussion

### 3.1. General

The syntheses of the Schiff base and its complexes may be represented by the following equation:

(3) MCH3COOH2·nH2O+H2LL⟶MLL+2CH3COOH+nH2OM=CuII,ZnII  n=2;  M=NiII,CoII  n=4.

**Figure 1:**
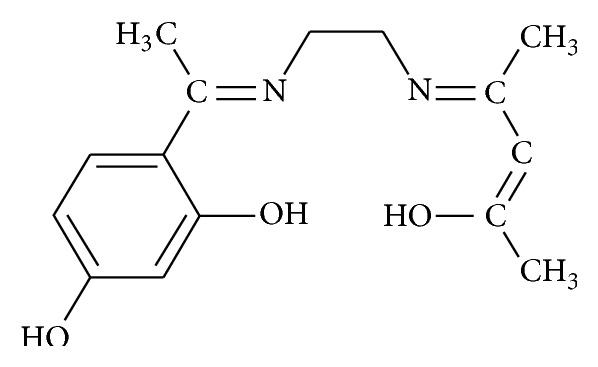
Structure of Schiff base ligand (H_2_LL).

**Figure 2:**
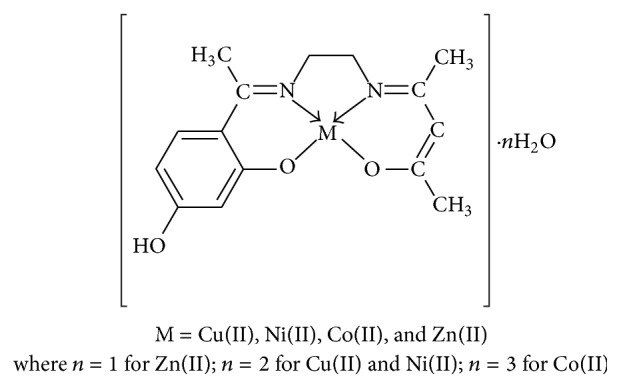
Proposed structure of metal complexes.

The structure of the ligand is shown in Figure 1 and the proposed structures for the complexes are shown in Figure 2. The obtained complexes are coloured powders, stable in air, and insoluble in water and other common solvents but are easily soluble in polar coordinating solvents such as DMF and DMSO. The physical characteristics, analytical data, and molar conductance data of ligand and metal complexes are given in Table 1 and are in good agreement with the proposed formulation. The observed molar conductance of the metal complexes solutions in DMF is consistent with the nonelectrolyte nature (3.22–4.27 *μ*Scm^−1^) of the complexes at room temperature [24].

### 3.2. Infrared Spectra

The relevant FTIR data for the ligands and metal complexes are given in Table 2. The infrared of H_2_LL shows characteristic bands at 3475 cm^−1^ that could be attributed to the phenolic hydroxyl group, and the characteristics absorption at 1612 cm^−1^ and 1243, 1271 cm^−1^ can be assigned to *ν*_(C=N)_ and *ν*_(C-O)_, respectively [5, 6]. The band at 3475 cm^−1^ was absent in the spectra of the complexes; this is indicative of the deprotonation and involvement of the phenolic hydroxyl group of the ligand in bond formation with the metal ions. The broad band at ~3400 cm^−1^ in the spectra of the Schiff base metal complexes is assigned to the *ν*_(OH)_ frequency of the coordinated H_2_O [16]. This is further supported by the upward shift in phenolic *ν*_(C-O)_ [15] to the extent of 23–30 cm^−1^ [25]. This shift further confirms the participation of the phenolic oxygen leading to the formation of C-O-M bond. The metal complexes show a broad band at ~3400 cm^−1^ and a new band at ~860 cm^−1^ that may be assigned to the stretching vibration and out of plane bending vibration of water molecules [16]. The strong band observed at 1612 cm^−1^ in the spectra of the free Schiff base ligand is a characteristic of the azomethine *ν*_(C=N)_ stretching vibrations. Upon complexation, this vibration underwent a shift to lower frequency 1584–1598 cm^−1^, indicating the bonding of unsaturated nitrogen of the azomethine group of H_2_LL to the metal ions [26]. This shift can further be explained by the donation of electrons from nitrogen to the empty d-orbitals of the metal ions [25]. Furthermore, some new bands observed between 502 and 536 cm^−1^ are attributed to *ν*_(M-N=C)_ and those within the band of 427–466 cm^−1^ are assigned to *ν*_(M-O)_ [27]. The FTIR spectra data confirmed the coordination of the imino nitrogen and phenolic oxygen atoms to the Zn, Cu, Ni, and Co ions.

### 3.3. Electronic Spectra Studies

**Figure 3:**
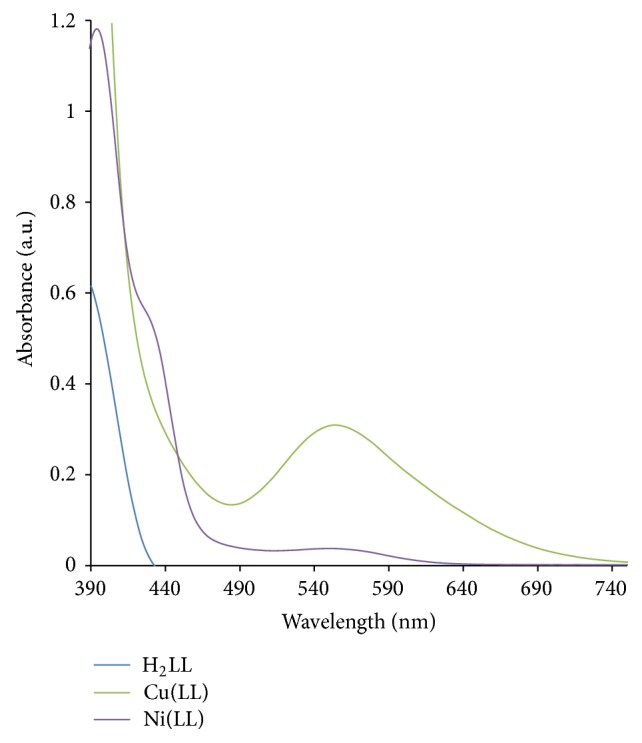
Electronic absorption spectra of H_2_LL, Cu(II), and Ni(II) complexes in DMF.

The UV-visible spectra of the Schiff base (H_2_LL) and its metal complexes were recorded in DMF solution at 298 K and presented in Figure 3. Relevant electronic spectra data are presented in Table 3. The UV-Vis spectra of H_2_LL showed two bands at 317 and 381 nm. The first band can be attributed to *π*-*π* ^∗^ transition within the aromatic ring, while the second band would be due to n-*π* ^∗^ transition within -C=N group. Upon complexation, n-*π* ^∗^ transition of ligand shifts to a longer wavelength; this indicates the coordination of ligand to metal [28]. The electronic spectrum of Co(II) complex of H_2_LL shows a CT band at 382 nm. The d-d absorption at 552 nm of less intensity indicates distorted tetrahedral environment of the ligand around the metal ion due to ^4^A_2_(F) → ^4^T_1_(P) transition [29]. Hence, Co(II) complex can be assigned distorted tetrahedral geometry. The electronic spectrum of Ni(II) complex exhibited a band at 394 nm attributable to charge-transfer transitions, L → M (LMCT), and two absorption bands at 436 and 564 nm which may be assigned to two spin allowed transitions, ^1^A_1g_ → ^1^A_2g_ and ^1^A_1g_ → ^1^B_1g_, respectively, characteristic of square-planar geometry around Ni(II) ion [30]. The observed electronic transitions and reddish-brown color of the complex further confirm square-planar geometry for Ni(II) complex [27]. Cu(II) complex of H_2_LL displayed band at 393 nm; this can be attributed to charge transfer, and the spectrum also showed d-d electronic transition at 556 nm which was assigned to ^2^B_1g_ → ^2^A_1g_ transition in square-planar geometry for the Cu(II) ion [6, 31]. Furthermore, the complex is nonelectrolyte as the molar conductance was found to be 3.91 *μ*Scm^−1^ in 10^−3 ^M in DMF as solvent in Table 1. The high-energy band in the region 411 nm in the zinc(II) complex is attributed to charge-transfer transitions L → M (LMCT), as d-d transition is not expected [16].

### 3.4. Powder XRD

**Figure 4:**
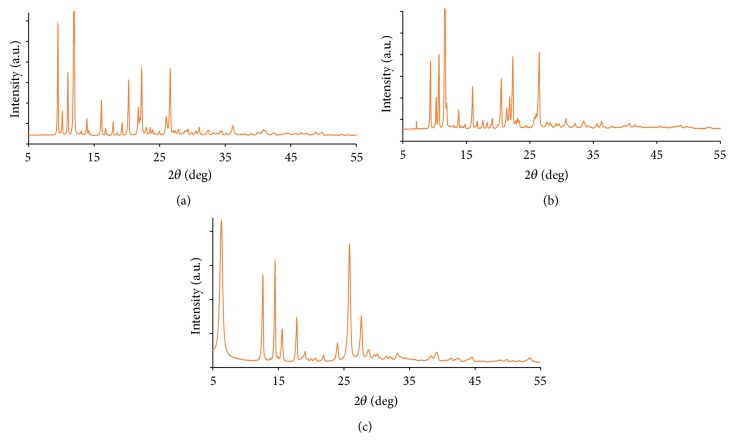
Powder XRD pattern of the (a) Cu(II), (b) Ni(II), and (c) Zn(II) complexes.

The X-ray diffraction of the metal complexes was scanned in the range 2*θ* = 0–60 at wavelength 1.5406 $\overset{´}{Å}$ as shown in Figure 4. The diffractogram and associated data depict the 2*θ* value for each peak, relative intensity, and interplanar spacing (*d*-values). The average crystallite size (*d*_XRD_) of the complexes was calculated using Scherer's formula [29, 32]:

(4) $$\begin{array}{c} d_{\text{XRD}}=\frac{0.9\lambda }{\beta \left(\cos\theta \right)}, \end{array}$$

where “*λ*” is the wavelength, “*β*” is the full width at half maxima, and “*θ*” is the diffraction angle.

XRD patterns of the metal complexes exhibited sharp crystalline peaks which indicate their crystalline phase. It can be observed that the diffractogram of the Schiff base differs from its metal complexes, which may be ascribed to the formation of a well-defined crystalline structure except Co(II) complex, which did not exhibit well-defined crystalline peaks indicating that the complex is amorphous [33]. The Ni(II), Cu(II), and Zn(II) Schiff base complexes have an average crystallite size of 46, 44, and 26 nm, respectively.

### 3.5. Antioxidant Activity

It is well known that reactive oxygen species (ROS) formed during biochemical processes in body system, such as superoxide anion, hydroxyl radical, and hydrogen peroxide, are vastly reactive and potentially damaging transient chemical species. The oxidative damages caused by ROS on lipids, proteins, and nucleic acids may generate various chronic diseases, such as coronary heart disease, atherosclerosis, cancer, and aging [30]. Hence, to prevent the free radical damage in the body, it is important to administer drugs that may be rich in antioxidants. The antioxidant assay study was carried out using different concentrations of the Schiff base and Zn(II), Ni(II), Cu(II), and Co(II) metal complexes with DPPH and ABTS radicals, while ascorbic acid (vitamin C), rutin, and butylated hydroxytoluene (BHT) were used as standards.

#### 3.5.1. DPPH Radical Scavenging Assay

**Figure 5:**
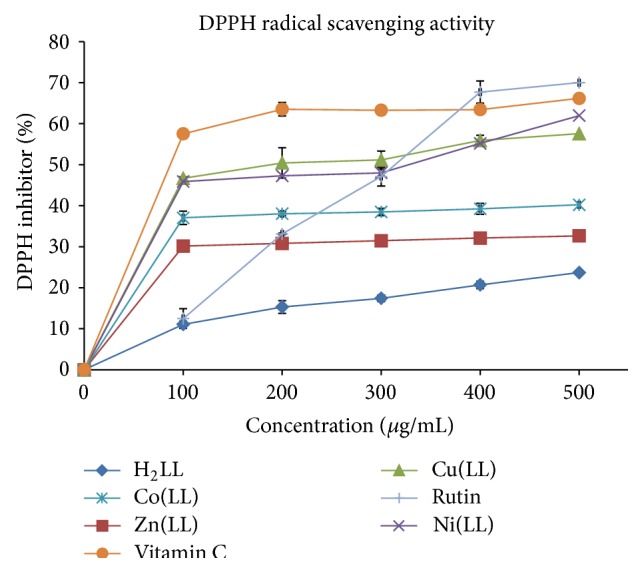
DPPH scavenging activity of H_2_LL Schiff bases and their metal complexes.

1,1-Diphenyl-2-picrylhydrazyl (DPPH) is a stable organic radical compound and its oxidative assay is used extensively in the quantification of radical scavengers capacity or hydrogen donors ability of samples. The antioxidant activities of Schiff base (H_2_LL), Zn(II), Ni(II), Cu(II), and Co(II) metal complexes together with the standards were assessed (Table 4) on the basis of the free radical scavenging effect of the stable DPPH free radical activity [34]. The examined changes in the free radical scavenging ability of the test samples on the basis of percent inhibition are presented in Figure 5. The DPPH scavenging activity of Schiff base metal complexes is significantly higher than that of free ligand (H_2_LL), indicating that this complex is a much better/stronger free radical scavenger and antioxidant than H_2_LL but lower when compared to ascorbic acid (vitamin C) and rutin as standards. Radical scavenging activity of metal complexes as well as the standards was increased in a dose-dependent manner, antioxidant ability of Schiff base (H_2_LL), increased significantly after chelation of transition metal ions (Figure 5). IC_50_ value of the tested samples is presented in Table 4 alongside with the correlation coefficient (*R* ^2^) values. The Cu(LL) and Ni(LL) complexes possess higher antioxidant potential (IC_50_) than rutin but lower than vitamin C (standard drugs). The order can be given as vitamin C > Cu(LL) > Ni(LL) > Co(LL) > rutin > Zn(LL) > H_2_LL with IC_50_ values as 1.92 ± 1.07 > 2.31 ± 1.54 > 2.34 ± 0.85 > 3.18 ± 0.96 > 2.52 ± 1.60 > 3.93 ± 1.64 > 6.47 ± 2.96 *μ*M. The oxidizing potentials of the samples are associated with the presence of compounds to exert actions by breaking the free radical chain via hydrogen atom donation [35]. Therefore, the results obtained from this study provide linkage to the use of the synthesized compounds in the treatment of pathological diseases arising from oxidative stress.

#### 3.5.2. ABTS Radical Scavenging Activity

**Figure 6:**
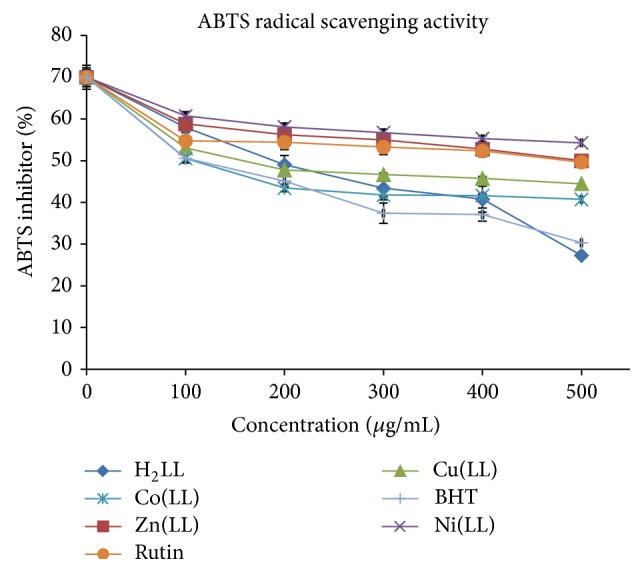
ABTS radical scavenger activity of H_2_LL and H_2_LL-M(II) complex.

An important attribute of antioxidants is the proton radical scavenging. A well-known protonated radical like 2,2′-azinobis-3-ethylbenzothiazoline-6-sulfonic acid (ABTS) has characteristic absorbance maxima at 734 nm which decreases with the scavenging of the proton radicals [36]. The Schiff base, Zn(II), Ni(II), Cu(II), and Co(II) metal complexes were moderate and effective scavengers of the ABTS radical (Figure 6) and this activity was comparable to those of rutin and BHT (Table 5) that are used as standard drugs. Lowest concentrations of the test samples were more effective in quenching ABTS^+^ radicals in the system. The scavenging of the ABTS^+^ radical by the Schiff base and its metal complexes was found to possess moderate to high activities relative to those of the standards (rutin and BHT). Co(LL) exhibited the highest activity with an IC_50_ of about 1.83 ± 1.08 *μ*M amongst the synthesized metal complexes. The ABTS radical scavenging ability of the tested compounds can be ranked in the order BHT > Co(LL) > H_2_LL > Cu(LL) > rutin > Zn(LL) > Ni(LL). The scavenging of the ABTS radical by the Schiff base (H_2_LL), Cu(II), Zn(II), Ni(II), and Co(II) metal complexes was found to be moderate compared to that of DPPH radical, indicating their potentials as chemotherapeutic agents for radicals chains terminator.

### 3.6. *In Vitro* Biological Activity

*In vitro* antibacterial screening of the Schiff base and its metal complexes (Table 5) was tested against three Gram-negative bacteria, namely,* Staphylococcus aureus* (ATCC 25923),* Streptococcus faecalis* (ATCC 29212), and* Bacillus cereus* (ATCC 10702), and three Gram-negative bacteria, namely,* Pseudomonas aeruginosa* (ATCC 19582),* Escherichia coli* (ATCC 25922), and* Shigella flexneri* (KZN), using disc diffusion method [21]. The compounds were tested at the concentrations 0.312–10 mg mL^−1^ in DMSO and compared with known antibiotics: Amoxicillin and Ciprofloxacin. The results in Table 6 shows that the metal complexes are more active than the free ligand and such enhanced activity of metal chelates is due to the lipophilic nature of the metal ions in complexes [15]. It also suggests that the complexes possess antibacterial activity inhibiting multiplication process of the microbes by blocking their active sites [29]. The ligand shows no antibacterial activity against all tested bacteria strains. The cobalt(II) complex shows slight effect* against S. faecalis, B. cereus,* and* E. coli*, while nickel(II) complex shows low antibacterial against all the stains with the exception of* S. aureus, P. aeruginosa,* and* S. flexneri*. The copper(II) complex exhibited low to higher bactericidal activities than other complexes against* S. faecalis, B. cereus, S. aureus, S. flexneri,* and* E. coli.*

The variation in the activity of different complexes against tested organisms depends on either the impermeability of cells of the microbes or difference in ribosomes of microbial cells [7]. The higher biological activity of metal complexes than that of the ligand (H_2_LL) can be explained on the basis of Overtone's concept and Tweedy's chelation theory [37]. On chelation, metal ion polarity is reduced to a greater extent due to the overlapping of the ligand orbital and partial sharing of positive charge of metal ion with donor groups [38]. Further, the delocalization of the *π*-electrons is increased over the whole chelate sphere and enhances the lipophilicity of the complex. The lipophilic nature of the central metal atom is also increased upon chelation, which subsequently favors the permeation through the lipid layer of cell membrane [6]. Notably, the normal cell process may be affected by the formation of hydrogen bond through the azomethine nitrogen atom with the active centres of cell constituents leading to interference with the cell wall synthesis [15, 25]. The bioactivity of the ligand and its complexes is found to be in the following order: Cu(II) > Co(II) > Ni(II) > Zn(II) > H_2_LL. The difference in antimicrobial activity is due to the nature of metal ions and also the cell membrane of the microorganisms.

## 4. Conclusion

In the present study, we synthesized new ligand of (4*E*)-4-[(2-{(*E*)-[1-(2,4-dihydroxyphenyl)ethylidene]amino}ethyl)imino]pentan-2-one, which was used in the preparation of Cu(II), Ni(II), Zn(II), and Co(II) complexes. The analytical data show that the metal ligand stoichiometry in all these complexes is 1 : 1. All the complexes are nonelectrolytes in DMF solution. The spectral data show that the synthesized ligand binds with metal ions in tetradentate through nitrogen atom of the azomethine and oxygen atoms of hydroxyl group of the 2′,4′-dihydroxyacetophenone beside the hydroxyl group of the carboxyl group of the acetylacetone, respectively. The* in vitro* biological evaluation of complexes against various pathogenic bacterial strains reveals that the metal complexes exhibited higher antimicrobial activity than the free ligand. Additionally, the compounds exhibited some antioxidant properties of scavenging free radicals. The results from DPPH and ABTS methods revealed that compounds are capable of donating electron or hydrogen atom and subsequently react with free radicals or terminate chain reactions in a dose-dependent pattern. The complexes show stronger scavenging effects than the ligand.

## Figures and Tables

**Table 1:** Physical characterization, analytical data, and molar conductance of the Schiff base ligand [H_2_LL] and its complexes.

Compounds	Empirical formula	M. Wt (g)	Color	Yield (%)	% found (Calcd)			Decomposition Temp, °C	Conductance (*µ*Scm^−1^)
					C	H	N		
H_2_LL	C_15_H_19_N_2_O_3_	275.32	Golden yellow	61.23	65.26 (65.44)	7.13 (6.96)	9.98 (10.17)	235	—
[Cu(LL)]·2H_2_O	C_15_H_21_N_2_O_5_Cu	372.89	Dark-purple	77.27	48.55 (48.32)	5.43 (5.68)	7.23 (7.51)	229	3.91
[Ni(LL)]·2H_2_O	C_15_H_21_N_2_O_5_Ni	368.03	Reddish-brown	76.20	49.11 (48.95)	5.52 (5.75)	7.44 (7.61)	191	4.47
[Co(LL)]·3H_2_O	C_15_H_23_N_2_O_6_Co	368.29	Darkish-brown	69.30	46.81 (46.64)	6.19 (6.00)	7.50 (7.25)	232	3.57
[Zn(LL)]·H_2_O	C_15_H_19_N_2_O_4_Zn	356.71	Lemon yellow	65.28	50.23 (50.51)	5.08 (5.37)	8.03 (7.85)	227	3.22

**Table 2:** FTIR spectral data of the Schiff base ligand [H_2_LL] and its metal complexes.

Compound	*ν*(OH)·*ν*(H_2_O)	*ν*(CH_3_/CH_2_)	*ν*(C=N)	*ν*(C=C)	*ν*(C-O)	*ν*(M-N)	*ν*(M-O)
H_2_LL	3475 sb	2928 w, 2911 w	1612 vs	1534 s, 1480 m	1243 s, 1271 s	—	—
[Cu(LL)]·2H_2_O	3399 mb	2975 s, 2902 m	1588 vs	1535 s, 1439 s	1241 s, 1180 m	522 m	466 m
[Ni(LL)]·2H_2_O	3346 mb	2975 s, 2900 m	1584 vs	1534 s, 1442 s	1242 s, 1180 m	502 m	465 w
[Co(LL)]·3H_2_O	3422 mb	2976 s, 2902 s	1598 vs	1542 m, 1461 m	1250 s, 1190 m	504 m	427 m
[Zn(LL)]·H_2_O	3417 sb	2974 m, 2901 m	1588 vs	1534 s, 1481 m	1266 s, 1242 s	536 m	436 s

s: strong; b: broad; v: very; m: medium; w: weak.

**Table 3:** Electronic absorption data and assignments of Schiff base ligand [H_2_LL] and its metal complexes.

Compounds		Electronic transition, *λ*_max⁡_ (nm, DMF)	Band assignments
H_2_LL	C_15_H_19_N_2_O_3_	317, 381	*π*-*π* ^∗^, n-*π* ^∗^
Cu(LL)	C_15_H_18_N_2_O_3_Cu	309, 360, 393, 556	*π*-*π* ^∗^, L → M (LMCT), ^2^B_1g_ → ^2^A_1g_
Ni(LL)	C_15_H_18_N_2_O_3_Ni	288, 308, 394, 436, 564	*π*-*π* ^∗^, L → M (LMCT), ^1^A_1g_ → ^1^A_2g_, ^1^A_1g_ → ^1^B_1g_
Co(LL)	C_15_H_18_N_2_O_3_Co	281, 382, 552	*π*-*π* ^∗^, L → M (LMCT), ^4^A_2_ (F) → ^4^T_1_ (P)
Zn(LL)	C_15_H_18_N_2_O_3_Zn	319, 361, 411	*π*-*π* ^∗^, *n*-*π* ^∗^, L → M (LMCT)

**Table 4:** DPPH scavenging capacities (IC_50_, *µ*M) of standard drugs and H_2_LL Schiff Bases and their metal complexes.

Compounds							
	H_2_LL	Cu(LL)	Ni(LL)	Co(LL)	Zn(LL)	Vitamin C^∗^	Rutin^∗^
IC_50_ ± SD	6.47 ± 2.96	2.31 ± 1.54	2.34 ± 0.85	3.18 ± 0.96	3.93 ± 1.64	1.92 ± 1.07	2.52 ± 1.60
*R* ^2^	0.909	0.992	0.977	0.968	0.969	0.978	0.768

(*n* = 3, *X*  ±  SEM); IC_50_: inhibitory concentration; *R* ^2^ = correlation coefficient. ^∗^Standards.

**Table 5:** ABTS radical scavenging activities (IC_50_, *µ*M) of standard drugs and H_2_LL Schiff bases and their metal complexes.

Compounds							
	H_2_LL	Cu(LL)	Ni(LL)	Co(LL)	Zn(LL)	Rutin^∗^	BHT^∗^
IC_50_ ± SD	1.86 ± 2.28	2.15 ± 1.85	3.66 ± 1.16	1.83 ± 1.08	3.08 ± 0.76	2.83 ± 1.84	1.64 ± 1.54
*R* ^2^	0.845	0.992	0.975	0.986	0.953	0.983	0.919

(*n* = 3, *X*  ±  SEM); IC_50_: inhibitory concentration; *R* ^2^: correlation coefficient. ^∗^Standards.

**Table 6:** Minimum inhibitory concentration values (mg/mL) of the Schiff base ligand [H_2_LL] and its metal complexes.

Compounds	Gram-positive bacteria			Gram-negative bacteria		
	S. *faecalis *	*B. cereus *	*S. aureus *	*P. aeruginosa *	*S. flexneri *	*E. coli *
H_2_LL	<10	10	<10	<10	<10	<10
Cu(LL)	5	2.5	5	<10	10	5
Ni(LL)	<10	10	5	10	<10	<10
Co(LL)	10	5	<10	<10	<10	10
Zn(LL)	<10	10	<10	<10	<10	10
Amoxicillin^a^	1.250	0.312	1.250	1.250	1.250	0.625
Ciprofloxacin^a^	0.312	0.312	0.312	0.312	0.312	0.312

^a^Standards.

## References

[1] Mishra N., Poonia K., Kumar D. (2013). An overview of biological aspects of Schiff base metal complexes. *International Journal of Advancements in Research & Technology*.

[2] Kumar S., Dhar D. N., Saxena P. N. (2009). Applications of metal complexes of Schiff bases—a review. *Journal of Scientific and Industrial Research*.

[3] Gaballa A. S., Asker M. S., Barakat A. S., Teleb S. M. (2007). Synthesis, characterization and biological activity of some platinum(II) complexes with Schiff bases derived from salicylaldehyde, 2-furaldehyde and phenylenediamine. *Spectrochimica Acta Part A: Molecular and Biomolecular Spectroscopy*.

[4] Genin M. J., Biles C., Keiser B. J. (2000). Novel 1,5-diphenylpyrazole nonnucleoside HIV-1 reverse transcriptase inhibitors with enhanced activity versus the delavirdine-resistant P236L mutant: lead identification and SAR of 3- and 4-substituted derivatives. *Journal of Medicinal Chemistry*.

[5] Malik S., Ghosh S., Mitu L. (2011). Complexes of some 3d-metals with a Schiff base derived from 5-acetamido-1,3,4-thiadiazole-2-sulphonamide and their biological activity. *Journal of the Serbian Chemical Society*.

[6] Alias M., Kassum H., Shakir C. (2014). Synthesis, physical characterization and biological evaluation of Schiff base M(II) complexes. *Journal of the Association of Arab Universities for Basic and Applied Sciences*.

[7] Mounika K., Pragathi A., Gyanakumari C. (2010). Synthesis*¸* characterization and biological activity of a Schiff base derived from 3-ethoxy salicylaldehyde and 2-amino benzoic acid and its transition metal complexes. *Journal of Scientific Research*.

[8] Gup R., Kirkan B. (2006). Synthesis and spectroscopic studies of mixed-ligand and polymeric dinuclear transition metal complexes with bis-acylhydrazone tetradentate ligands and 1,10-phenanthroline. *Spectrochimica Acta Part A: Molecular and Biomolecular Spectroscopy*.

[9] Usha A. K., Chandra S. (1992). Pd(II), Pt(II), Rh(III), Ir(III) and Ru(III) Complexes of n-Pentyl and n-Hexyl Ketone Thiosemicarbazones. *Synthesis and Reactivity in Inorganic and Metal-Organic Chemistry*.

[10] Wang W., Zeng F.-L., Wang X., Tan M.-Y. (1996). A study of an oxovanadium(V) complex with a tridentate Schiff base ligand. *Polyhedron*.

[11] Zishen W., Zhiping L., Zhenhuan Y. (1993). Synthesis, characterization and antifungal activity of glycylglycine Schiff base complexes of 3d transition metal ions. *Transition Metal Chemistry*.

[12] Shivakumar K., Shashidhar, Reddy P. V., Halli M. B. (2008). Synthesis, spectral characterization and biological activity of benzofuran Schiff bases with Co(II), Ni(II), Cu(II), Zn(II), Cd(II) and Hg(II) complexes. *Journal of Coordination Chemistry*.

[13] Cotton F. A., Wilkinson G. (1988). *Advanced Inorganic Chemistry*.

[14] Tümer M., Köksal H., Serin S., Digrak M. (1999). Antimicrobial activity studies of mononuclear and binuclear mixed-ligand copper(II) complexes derived from Schiff base ligands and 1,10-phenanthroline. *Transition Metal Chemistry*.

[15] Shelke V. A., Jadhav S. M., Patharkar V. R., Shankarwar S. G., Munde A. S., Chondhekar T. K. (2012). Synthesis, spectroscopic characterization and thermal studies of some rare earth metal complexes of unsymmetrical tetradentate Schiff base ligand. *Arabian Journal of Chemistry*.

[16] Osowole A. A., Kolawole G. A., Fagade O. E. (2005). Synthesis, physicochemical, and biological properties of nickel(II), copper(II), and zinc(II) complexes of an unsymmetrical tetradentate Schiff base and their adducts. *Synthesis and Reactivity in Inorganic, Metal-Organic and Nano-Metal Chemistry*.

[17] Arulpriya P., Lalitha P., Hemalatha S. (2010). *In vitro* antioxidant testing of the extracts of *Samanea saman (Jacq.) Merr*. *Der Chemica Sinica*.

[18] Scherer R., Godoy H. T. (2009). Antioxidant activity index (AAI) by the 2,2-diphenyl-1-picrylhydrazyl method. *Food Chemistry*.

[19] Al-Amiery A. A., Kadhum A. A. H., Mohamad A. B. (2012). Antifungal and antioxidant activities of pyrrolidone thiosemicarbazone complexes. *Bioinorganic Chemistry and Applications*.

[20] Adedapo A. A., Jimoh F. O., Koduru S., Afolayan A. J., Masika P. J. (2008). Antibacterial and antioxidant properties of the methanol extracts of the leaves and stems of *Calpurnia aurea*. *BMC Complementary and Alternative Medicine*.

[21] Parekh J., Inamdhar P., Nair R., Baluja S., Chanda S. (2005). Synthesis and antibacterial activity of some Schiff bases derived from 4-aminobenzoic acid. *Journal of the Serbian Chemical Society*.

[22] Afolayan A. J., Meyer J. J. M. (1997). The antimicrobial activity of 3,5,7-trihydroxyflavone isolated from the shoots of *Helichrysum aureonitens*. *Journal of Ethnopharmacology*.

[23] National Committee for Clinical Laboratory Standards (2004). Methods for dilution antimicrobial susceptibility tests for bacteria that grow aerobically. *Approved Standard Document*.

[24] Geary W. J. (1971). The use of conductivity measurements in organic solvents for the characterisation of coordination compounds. *Coordination Chemistry Reviews*.

[25] Neelakantan M. A., Marriappan S. S., Dharmaraja J., Jeyakumar T., Muthukumaran K. (2008). Spectral, XRD, SEM and biological activities of transition metal complexes of polydentate ligands containing thiazole moiety. *Spectrochimica Acta Part A: Molecular and Biomolecular Spectroscopy*.

[26] Ajibade P. A., Kolawole G. A., O'Brien P., Helliwell M., Raftery J. (2006). Cobalt(II) complexes of the antibiotic sulfadiazine, the X-ray single crystal structure of [Co(C_10_H_9_N_4_O_2_S)_2_(CH_3_OH)_2_]. *Inorganica Chimica Acta*.

[27] Abd-Elzar M. M. (2001). Spectroscopic characterization of some tetradentate Schiff bases and their complexes with Nickel, Copper and Zinc. *Journal China Chemica Society*.

[28] Kaushal R., Thakur S. (2013). Syntheses and biological screening of schiff base complexes of titanium(IV). *Chemical Engineering Transactions*.

[29] Joseyphus R. S., Nair M. S. (2008). Antibacterial and antifungal studies on some Schiff base complexes of zinc(II). *Mycobiology*.

[30] Akila E., Usharani M., Rajavel R. (2013). Metal (II) complexes of bioinorganic and medicinal relevance: antibacterial, antioxidant and dna cleavage studies of tetradentate complexes involving o, n-donor environment of 3, 3′-dihydroxybenzidine-based schiff bases. *International Journal of Pharmacy and Pharmaceutical Sciences*.

[31] Lever A. B. P. (1980). *Inorganic Electronic Spectroscopy*.

[32] Souaya E. R., Hanna W. G., Ismail E. H., Milad N. E. (2000). Studies on some acid divalent-metal nitrilotriacetate complexes. *Molecules*.

[33] Arish D., Nair M. S. (2010). Synthesis, characterization, antimicrobial, and nuclease activity studies of some metal Schiff-base complexes. *Journal of Coordination Chemistry*.

[34] Braca A., Sortino C., Politi M., Morelli I., Mendez J. (2002). Antioxidant activity of flavonoids from *Licania licaniaeflora*. *Journal of Ethnopharmacology*.

[35] Hernández J. A., Jiménez A., Mullineaux P., Sevilla F. (2000). Tolerance of pea (*Pisum sativum* L.) to long-term salt stress is associated with induction of antioxidant defences. *Plant, Cell and Environment*.

[36] Mathew S., Abraham T. E. (2006). *In vitro* antioxidant activity and scavenging effects of *Cinnamomum verum* leaf extract assayed by different methodologies. *Food and Chemical Toxicology*.

[37] Tweedy B. G. (1964). Plant extracts with metal ions as potential antimicrobial agents. *Phytopathology*.

[38] Thangadurai T. D., Natarajan K. (2001). Mixed ligand complexes of ruthenium(II) containing *α*,*β*-unsaturated-*β*-ketoamines and their antibacterial activity. *Transition Metal Chemistry*.

